# Identification of an Intrinsic Determinant Critical for Maspin Subcellular Localization and Function

**DOI:** 10.1371/journal.pone.0074502

**Published:** 2013-11-21

**Authors:** Sijana H. Dzinic, Alexander Kaplun, Xiaohua Li, Margarida Bernardo, Yonghong Meng, Ivory Dean, David Krass, Paul Stemmer, Namhee Shin, Fulvio Lonardo, Shijie Sheng

**Affiliations:** 1 Department of Pathology, Wayne State University School of Medicine, Detroit, Michigan, United States of America; 2 The Tumor and Microenvironment Program of the Barbara Ann Karmanos Cancer Institute, Detroit, Michigan, United States of America; 3 The Institute of Environmental Health Sciences, Proteomics Core Facility, Wayne State University, Detroit, Michigan, United States of America; Louisiana State University Health Sciences center, United States of America

## Abstract

Maspin, a multifaceted tumor suppressor, belongs to the serine protease inhibitor superfamily, but only inhibits serine protease-like enzymes such as histone deacetylase 1 (HDAC1). Maspin is specifically expressed in epithelial cells and it is differentially regulated during tumor progression. A new emerging consensus suggests that a shift in maspin subcellular localization from the nucleus to the cytoplasm stratifies with poor cancer prognosis. In the current study, we employed a rational mutagenesis approach and showed that maspin reactive center loop (RCL) and its neighboring sequence are critical for maspin stability. Further, when expressed in multiple tumor cell lines, single point mutation of Aspartate^346^ (D^346^) to Glutamate (E^346^), maspin^D346E^, was predominantly nuclear, whereas wild type maspin (maspin^WT^) was both cytoplasmic and nuclear. Evidence from cellular fractionation followed by immunological and proteomic protein identification, combined with the evidence from fluorescent imaging of endogenous proteins, fluorescent protein fusion constructs, as well as bimolecular fluorescence complementation (BiFC) showed that the increased nuclear enrichment of maspin^D346E^ was, at least in part, due to its increased affinity to HDAC1. Maspin^D346E^ was also more potent than maspin^WT^ as an HDAC inhibitor. Taken together, our evidence demonstrates that D^346^ is a critical *cis*-element in maspin sequence that determines the molecular context and subcellular localization of maspin. A mechanistic model derived from our evidence suggests a new window of opportunity for the development of maspin-based biologically competent HDAC inhibitors for cancer treatment.

## Introduction

Tumor suppressor maspin is a member of Clade B serine protease inhibitor (serpin) superfamily, but has been shown to have deviant biological functions and molecular modes of action [1], [2]. Earlier structural and experimental evidence suggests that maspin does not inhibit an active serine protease [3]–[5]. To date, we have identified only two circumstantial serine protease targets of maspin: the zymogen form of urokinase type plasminogen activator (pro-uPA) [6]–[8] and the single-chain tissue-type plasminogen activator (sc-tPA) that is associated with fibrinogen [1]. Our laboratory also discovered that endogenous maspin binds to and inhibits the activity of histone deacetylase 1 (HDAC1) [7], [9], which is a major nuclear deacetylase of class I that is up-regulated in many types of cancers [2]. To our knowledge, maspin is the only endogenous polypeptide inhibitor of HDAC1 identified thus far. Homozygous deletion of either the maspin gene or the HDAC1 gene results in embryonic lethality in murine models, signifying the equally vital roles of maspin and HDAC1 in embryogenesis [10], [11]. As compared to pharmacological HDAC inhibitors, maspin regulates a much smaller cluster of HDAC target genes, all being implicated in controlling epithelial differentiation [12]. These findings provided a functional connection between maspin and a better differentiated phenotype as well as better prognosis of human cancer [13].

Maspin is an epithelial-specific gene shown to be differentially regulated in the progression of many types of solid tumors including prostate [14], [15], breast [16], [17] and lung [18], [19]. The differential regulation of maspin occurs not only at the level of expression, but also at the level of subcellular distribution. In accordance with the spectrum of its possible molecular targets and overall tumor suppressive activities, maspin is known to be a secreted and cell surface-associated protein [20]. In normal epithelial tissues, the intracellular maspin is predominantly nuclear. Accumulated clinical evidence in lung [19], [21], breast [22] and ovarian [23], [24] carcinoma demonstrates that nuclear retention of maspin is correlated with better overall patient survival [14]. Conversely, the shift in subcellular localization of maspin from the nucleus to the cytoplasm is associated with a gain of function during tumor progression [19].

In a recent study, the expression of bioengineered maspin targeted for nuclear exclusion failed to exert tumor suppressive effects both *in vitro* and *in vivo* [25], [26]. This evidence supports the significance of maspin translocation from the nucleus to cytoplasm in tumor progression. However, it is important to note that maspin does not have any of the currently known intrinsic “address tags” such as nuclear localization sequence (NLS), nuclear export sequence (NES) or secretory leader sequence (SLS) [27]. To date, the molecular mechanisms that control maspin trafficking and maspin nuclear localization, in particular, are unknown. As a 42 kDa protein, maspin could passively diffuse through the nuclear envelope [28]. However, considering the distinct subcellular localization of maspin at different epithelial dedifferentiation states and the evidence that maspin is not mutated in tumor progression, we speculate that maspin subcellular localization may be actively controlled by its molecular partnership that is subjected to modulations by pathological signals. In parallel, it is reasonable to hypothesize that intracellular maspin trafficking also depends on key *cis*-elements in maspin primary sequence. To this end, maspin contains a reactive center loop (RCL) sequence, as expected based on its overall alignment with members of the serpin superfamily. Although the most variable and deviant among serpins [4], [5], [29], the RCL of maspin is essential for its biochemical effects on the serine protease-like targets [1], [6], [7], and its biological effects on tumor cell motility and invasion [15], [20], [30]. Based on several known serpin structures including that of maspin, amino acid residues in close vicinity to RCL may play an important role in the overall affinity for the target proteins. Interestingly, it is noted that at the C-terminal end of the RCL sequence of maspin is an Aspartate 346 (D^346^), which is unique among all serpin homologs and maspin orthologs.

In this study, we report that a conservative substitution of maspin D^346^ by glutamic acid (E) resulted in its dominant nuclear distribution and increased interaction with HDAC1 in multiple cancer cell lines. Our data led to a hypothetical model that helps explain how maspin nuclear retention and its tumor suppressive competency may be bypassed in tumor progression. These novel insights may guide future development of maspin-based biologically competent HDAC inhibitors for cancer treatment.

## Materials and Methods

### Cell Culture, Reagents and Antibodies

Immortalized normal human epithelial cells from prostate (CRL2221), breast (MCF10A) and lung (BEAS2B), human prostate carcinoma cell lines (DU145 and PC3) and human lung carcinoma cell line H1299 were purchased from American Type Culture Collection (ATCC, Manassas, VA). All media and media supplements were purchased from Life Technologies™ (Grand Island, NY) unless stated otherwise. CRL2221 cells were grown in Keratinocyte Serum-Free Medium with supplied growth factors, MCF-10A cells were maintained in Dulbecco’s modified Eagle’s medium (DMEM)/F12 with additional supplements [7] and BEAS 2B cells were cultured in LHC 8 media [19]. Carcinoma cell lines were maintained in RPMI 1640 media containing FBS from Hyclone (South Logan, UT; 5% for DU145; 10% for PC3 and H1299) [6]. All cells were cultures at 37°C with 6.5% CO_2_.

We used the following reagents: MG132 (#474790, Calbiochem, La Jolla, CA), Leptomycin B (#L2913-5UG, Sigma Aldrich, St. Louis, MO), goat serum (# ab7481, Abcam, Cambridge, MA), protein A/G agarose beads (# sc2003, Santa Cruz, Santa Cruz, CA), normal mouse IgG (#sc52003, Santa Cruz), normal rabbit IgG (# sc3888, Santa Cruz) and DAPI (#10236276001, Roche Applied Science, Foster City, CA). Primary antibodies used include: maspin (#554292, BD Pharmingen, San Jose, CA), HDAC1 (#06720, Millipore, Grand Island, NY), poly (ADP-ribose) polymerase (PARP, #04–575, Millipore), acetylated histone 3 at lysine 9 (Acetyl H3 K9, #07352, Millipore), Lamin B (#ab16048, Abcam), GAPDH (#9484, Abcam), β tubulin (#ab6046, Abcam), and glucose regulated protein 78 (#sc13968, GRP78, Santa Cruz). The secondary antibodies used include: anti mouse-HRP conjugated and anti-rabbit-HRP conjugated (#NXA931 and #NA934, respectively, from GE Health Care, Fairfield, CT) and fluorescent dyes, Alexa Fluor 488 and Alexa Fluor 594 (#A11029 and #A21203, respectively, from Life Technologies™).

### Adenoviral Expression of Recombinant Maspin

Maspin recombinant proteins were generated as previously described [7]. Briefly, to generate truncation mutants maspin^1–323^, maspin^1–340^ and maspin^1–348^, the C-terminus of maspin after designated last amino acid residue in the pVL1393/maspin template vector [31] was fused in frame to a translation-stop codon using the following PCR primers. For maspin^1–323^: 5′-CTGAAGATGGTGGGGATTCCTAAGAGGTGCCAGGAGCACGG-3′, and 5′-CCGTGCTCCTGGCACCTCTTAGGAATCCCCACCATCTTCAG-3′; for maspin^1–340^: 5′-CCATAGAGGTGCCAGGAGCACGGTAACTGCAGCACAAGG-3′, and 5′-CCTTGTGCTGCAGTTACCGTGCTCCTGGCACCTCTGTGG-3′; for maspin^1–348^: 5′-CCTGCAGCACAAGGATGAATTGTAAGCTGACCATCCC-3′, and 5′-GGGATGGTCAGCTTACAATTCATCCTTGTGCTGCAGG-3′. Using the Exsite PCR-Based Site-Directed Mutagenesis kit we substituted Aspartate (D) 346 in the pVL1393/maspin template with Glutamic acid (E) to generate maspin^D346E^ mutant. The PCR primers for mutagenesis were 5′-CGGATCCTGCAGCACAAGGAAGAATTTAATGCTGACCATCCC-3′ and 5′-GGGATGGTCAGCATTAAATTCTTCCTTGTGCTGCAGGATCCG-3′. The cDNAs encoding maspin mutants were sequence-verified and sub-cloned into the pAdenoVator-CMV5 vector (Q-Bio Gene, Carlsbad, CA) as described [7]. The resulting plasmids were purified and used to generate the recombinant adenoviral DNA and the adenovirus using an established procedure [7]. Adenoviral titer was determined using the Quick Titer™ Adenovirus Titer ELISA kit according to the manufacturer’s instructions (Cell Biolabs, Inc., San Diego, CA). For routine *in vitro* infections, adenovirus was added to 24 hrs-old cell culture at the multiplicity of infection (MOI) of 30 (DU145 cells) or 20 (H1299 cells). For subsequent functional and biological assays, the cells were used three days post infection, unless stated otherwise, since the expression of recombinant proteins was at its maximum.

### Constructs for Fusion Proteins

The constructs for fluorescence-tagged maspin and HDAC1 were made as follows: for maspin-green fluorescent protein (GFP) chimera the coding region of human maspin cDNA was PCR amplified from pBluescript-maspin [16], [32] inserting SacI restriction sites. The amplified fragment was sub-cloned into pEGFP-N1 mammalian expression vector from Clontech (Mountain View, CA). For the construction of HDAC1-red fluorescent protein (RFP) chimera, mRFP was PCR amplified from pmRFP1-N1 vector and inserted into pcDNA3.1-HDAC1 (Addgene repository, plasmid 13820) and ligated using EcoRI. For this reason, an additional N-terminal EcoRI site had to be eliminated first by site-directed mutagenesis (QuikChange Lightning Site-Directed Mutagenesis Kit, Agilent Technologies, Santa Clara, CA). The constructs for bimolecular fluorescence complementation (BiFC) [33] were generously provided by Dr. Kerppola (University of Michigan, Ann Arbor, MI): BiFC bJunYN, BiFC bFosYC and BiFC bFosYC Δ179–193 that utilize yellow fluorescence protein (YFP) as a reporter. Mutagenesis in BiFC MaspinYC was performed using QuikChange Lightning Site-Directed Mutagenesis Kit (Agilent Technologies) to replace bFos with maspin in BiFC bFosYC plasmid and bJun with HDAC1 in BiFC bJunYN plasmid. The resulting constructs are designated as BiFC maspinYC and BiFC HDAC1YN, respectively.

### Transient Transfection

Cells grown in 6 well plates at 70% confluence were transfected or co-transfected with 1 µg of plasmid DNA for maspin-GFP and/or HDAC1-RFP, using the X-tremeGENE 9 DNA transfection reagent (Roche Applied Science, Indianapolis, IN). Fluorescence imaging of live cells expressing GFP- and/or RFP-fusion proteins was acquired 40–48 h after transient transfection using the Leica DM IRB fluorescence microscope. For transfection using the BiFC constructs, the experiments were performed as outlined by Kerppola [33], allowing the maturation of the fluorescent complexes for 24 hrs at 30°C prior to live cell imaging. In BiFC experiments, co-transfection with BiFC bJunYN and BiFC bFosYC, and the co-transfection with BiFC bJunYN and BiFC bFosYC Δ179–193 were used as positive and negative controls, respectively.

### Immunofluorescence Staining, Confocal and Live Cell Imaging

Cells grown in 8-well chamber slides (#154534, Thermo Fisher Scientific, Hudson, NH) to 70% confluence were fixed with 4% paraformaldehyde (15 min at room temperature (RT)), and permeabilized with 100% ice cold methanol (10 min at −20°C). The slides were incubated with 10% normal goat serum in PBS for 1 hr, and incubated with anti-maspin (1∶100) antibody alone or in a combination with either anti-lamin B (1∶50), anti-HDAC1 (1∶50) or anti-GRP78 (1∶50) at 4°C overnight. Cells were washed and incubated for 2 hrs at room temperature (RT) with Alexa Fluor 488 (1∶500) alone or in combination with Alexa Fluor 594 (1∶500). The nuclei were counterstained with DAPI. Life cell imaging of transiently transfected cells was performed using the Leica Fluorescent microscope. The confocal imaging was assisted by the Microscopy, Imaging and Cytometry Resources Core at Karmanos Cancer Institute, Wayne State University School of Medicine.

### Cellular Fractionation, Immunoprecipitation (IP) and Protein Identification by Mass Spectrophotometry (MS)

For identification and quantification of maspin in the cytosolic and nuclear compartments, cells were fractioned as previously described [34] or by using the Subcellular Protein Fractionation Kit (Thermo Fisher Scientific, Rockford, IL) according to the manufacturer’s instructions. Fractionated proteins were subjected to IP. Briefly, to block nonspecific background, protein A/G-agarose beads, at a final concentration of 5% (v/v), were incubated with 500 µg of total lysate (or fractionated cell lysates) in IP buffer as previously described [34]. The resulting mixture was centrifuged at 10,000×g for 30 s, and the harvested supernatant was incubated with 5 µg of maspin antibody or 5 µg of pre-immune mouse IgG with gentle agitation at 4°C overnight. Protein A/G beads were then added to a final concentration of 2.5% (v/v). The mixture was further incubated for 2 hrs at RT with gentle agitation and centrifuged at 10,000×g for 30 s. The harvested beads were washed four times with IP buffer and one time with PBS, re-suspended with SDS sample buffer, heat denatured, and subjected to SDS-PAGE. The proteins resolved by SDS-PAGE were either profiled by western blotting or by MS/proteomic analysis for definitive identification and quantification. For MS/proteomic analysis, SDS-PAGE gels were stained with Sypro Ruby (#S12000, Life Technologies™) and washed. Gel slices were reduced with DTT and alkylated with IAA prior to digesting proteins with trypsin as previously described [11]. Peptides extracted from the gels were identified by LC-MS/MS on a Q Exactive OrbiTrap system and quantified by spectral counting. Data analysis was performed using Proteome Discoverer 1.3, which incorporated the Mascot algorithm (Matrix Science). The UniProt human database was used and a reverse decoy protein database was run simultaneously for false discovery rate (FDR) determination. Secondary analysis was performed using Scaffold (Proteome Software). A fixed modification of +57 on cysteine (carbamidomethylation) and variable modifications of +16 on methionine (oxidation) and +42 on protein n-terminus (acetylation) were included in the search. Minimum protein identification probability was set at ≥95% with 2 unique peptides.

### Quantitative Real-time PCR (q-RT-PCR)

Total RNA was extracted (RNeasy Mini kit, Qiagen Valencia, CA,), and reverse-transcribed (iScript cDNA synthesis kit, Bio-Rad, Irvine, CA). The mRNA of the following genes was quantified using the following pairs of primers: AKR1C2 (5′-GTAAAGCTCTAGAGGCCGT-3′ and 5′-CTGGTCGATGGGAATTGCT-3′), Fst (5′-GTTTTCTGTCCAGGCAGCTCCAC-3′ and 5′-GCAAGATCCGGAGTGCTTTACT-3′), Glrx (5′-GGGAGCAAGAACGGTGCCTCG-3′ and 5′-ATCTGTGGTTACTGCAGAGCTCCA-3′), Kcnk1 (5′-GCACGGTGTGGCCATAACCTGT-3′ and 5′-GCCTCGGGCAACTGGAACTGG-3′), Lancl1 (5′-TGGCGCCCCTGGGGTAATCT-3′ and 5′-ACCATTCAGCAAACTTACAGGCCC-3′), Mx1 (5′-CCTATCACCAGGAGGCCAGCAAGC-3′ and 5′-TTCCGCTTGTCGCTGGTGTCG-3′), Nell2 (5′-ATGCCTGAATGGAACCATCCAGTGTG-3′ and 5′-TCGACAGCTACAAACAGCCCTGT-3′), Nmu (5′-CAGCCTCAGGCATCCAACGCA-3′ and 5′-GGAACGAGCTGCAGCAACGGA-3′), Tspan7 (5′-GGGTTGTTATGATCTGGTAACTAGTTTCATGGAGAC-3′ and 5′-GCCAGCAGCATGCCAATTAACTGG-3′), and Esrp1 (5′-CCAAGAAGAATGTACTATTACCTGAATGC-3′ and 5′-ACCTCGTGCCCTGACTACGGT-3′). RT-PCR cycle threshold of each gene was normalized using the GAPDH (5′-ATCACCATCTTCCAGGAGCGA-3′ and 5′-GCCAGTGAGCTTCCCGTTCA-3′) as an internal reference.

### Miscellaneous Procedures

Protein concentration was determined using the Pierce BCA Protein Assay Kit (#23225, Thermo Scientific). Western blotting analyses were carried out as described [7]. To characterize the proliferation kinetics of adenovirus-infected cells, the cells (1×10^4^/well) were seeded in a 6 well plate in duplicates. The next day, cells were infected with adenovirus and subsequently counted on days, 3, 6, 8, 10 and 13 using the Coulter Z1 particle counter (Beckman Coulter, Fullerton, CA) [7].

## Results

### Construction and Expression of Rational Maspin Mutants

**Figure 1:**
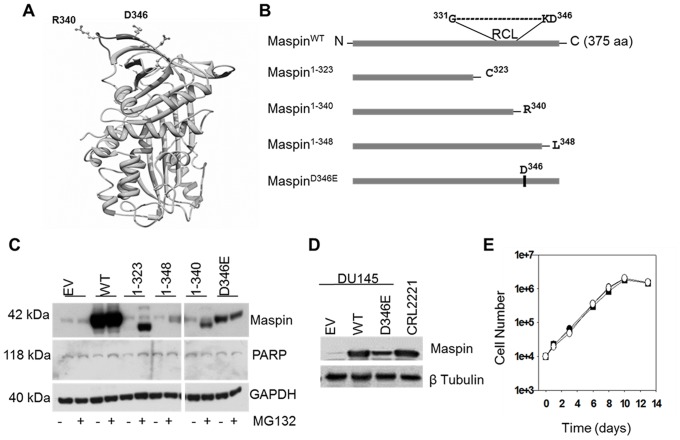
Construction and expression of rational maspin mutants. (**A**) Ribbon representation of human maspin generated by UCSF Chimera (http://www.cgl.ucsf.edu/chimera/). Relative positions of reactive center loop (RCL) p′ site R^340^ and neighboring D^346^ residue are depicted as ball and stick model. (**B**) Schematic illustration of the wild type human maspin and four rational maspin mutants. Relative positions of RCL and D^346^ in a primary maspin sequence are depicted. The full-length, wild type maspin was denoted as maspin^WT^, the truncation mutants are maspin^1–323^ (C-terminal deletion starting 16 amino acids upstream of RCL), maspin^1–340^ (C-terminal deletion after R^340^) and maspin^1–348^ (C-terminal deletion after L^348^) and the point mutant is maspin^D346E^. (**C**) Western blot of maspin^WT^ and four rational mutants re-expressed in prostate cancer cell line DU145 by adenoviral vector (multiplicity of infection, MOI = 30) in the presence or absence of proteasome inhibitor MG132 (5 µM, 6 hrs treatment). Infection of DU145 cells by adenovirus empty vector (EV) was used as a negative control. PARP was used to monitor cell viability after infection and MG132 treatment. GAPDH was used as a loading control. (**D**) Western blot of recombinant maspin in DU145 cells three days post-infection relative to the expression of endogenous maspin by CRL2221 cells. β tubulin was used as a loading control. (**E**) Growth curves of infected DU145 cells expressing either maspin^WT^ (•), maspin^D346E^ (○) or empty vector control (▪).

To investigate whether maspin RCL sequence and its neighboring regions, including the D^346^ residue, play a role in dictating maspin subcellular localization and function, we used the site directed mutagenesis approach to generate maspin mutants. Considering the exposed regions near maspin RCL, as shown by the ribbon structure of maspin resolved by crystallography [29], [35] (**Figure 1A**), our scheme for rational site-directed mutagenesis included maspin^1–323^, maspin^1–340^, maspin^1–348^ and maspin^D346E^ (**Figure 1B**). Previously, we successfully used an adenoviral expression system for endogenous expression of wild type maspin (maspin^WT^) [7] in DU145 cells that expresses a negligible level of endogenous maspin [6]. This system was used in the current study to express both wild type maspin (maspin^WT^) and maspin mutants. The adenovirus encoding empty vector was used as a negative control.

In our hands, maspin^WT^ was expressed as early as the second day after adenoviral infection, peaked at 3–5 days post-infection and remained steady up to 15 days post-infection (data not shown). It was noted that when used at the same MOI, adenoviruses encoding different maspin mutants resulted in an unequal level of protein production (**Figure 1C** **)**, with maspin^WT^ expressed at the highest level, followed by the maspin^D346E^. To test whether maspin mutants were unstable and, thus, subjected to proteasome-mediated degradation, cells were treated with a low dose of proteasome inhibitor MG132 for 6 hrs on the third day after infection. Judging by the integrity of PARP on western blot, the cell viability was not affected by adenoviral infection or MG132 treatment. While there was no net increase in the expression of maspin^WT^ and maspin^D346E^, MG132 treatment increased the level of detectable maspin^1–323^, maspin^1–340^ and maspin^1–348^. These data suggest that the RCL sequence and its neighboring regions are crucial for maspin stability. Regardless of treatment, maspin^D346E^ was expressed as a stable protein suggesting that the D^346^ to E^346^ conservative substitution did not affect the backbone of the maspin structure. We subsequently focused on the comparison of maspin^WT^ and maspin^D346E^. We also modified the infection MOI for each mutant to equalize the level of protein expression and made sure that the level of maspin^WT^ or maspin^D346E^ expressed in infected DU145 cells did not exceed the level of maspin endogenously expressed by normal immortalized prostate epithelial cells CRL2221 (**Figure 1D**). Consistent with the previous data with stably transfected DU145 cells [7], maspin expression by adenovirus did not affect cell proliferation (**Figure 1E**). Similarly, the expression of maspin^D346E^ did not exert additional effects on the exponential growth of tumor cells. Like maspin^WT^, maspin^D346E^ expressed by adenovirus did not cause cell death over a period as long as 14 days (**Figure 1E**).

### Subcellular Localization of Maspin^WT^ and Maspin^D346E^

**Figure 2:**
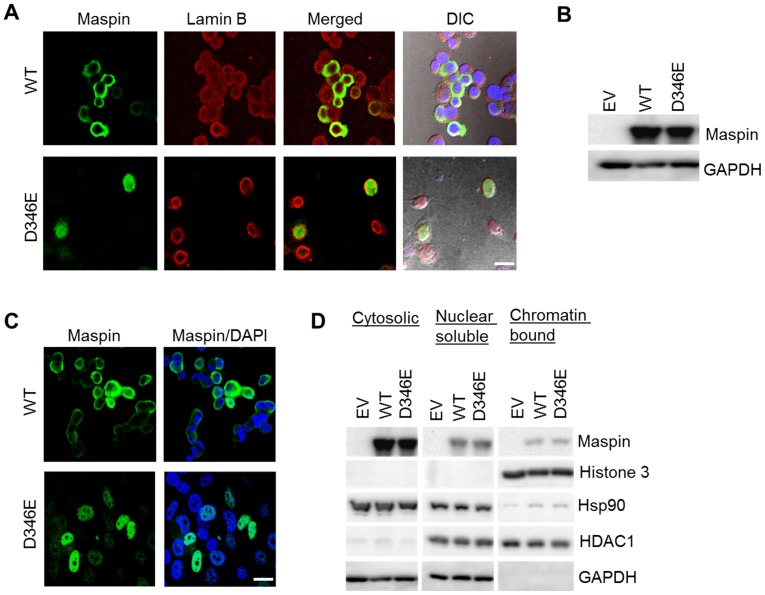
Subcellular localization of maspin^WT^ and maspin ^D346E^. (**A**) Confocal immunofluorescence imaging of maspin (green) and nuclear envelope marker lamin B (red) in DU145 cells after infection. (**B**) Western blot of recombinant maspin in H1299 cells 3 days post-infection with adenoviral vector (MOI = 20). GAPDH was used as a loading control. (**C**) Confocal immunofluorescence imaging of recombinant maspin in H1299 after adenoviral infection. Nuclei were counterstained with DAPI. Scale bars = 20 µm. (**D**) Western blotting of maspin, histone 3, Hsp90, HDAC1 and GAPDH in fractionated lysates of infected H1299 cells. ascertain the differential subcellular distributions of maspin^D346E^ and maspin^WT^, H1299 cells expressing recombinant maspin^WT^ or maspin^D346E^ were fractionated. As shown in **Figure 2D**, western blotting of HDAC1, histone 3, and Hsp90 plus GAPDH, demonstrated the purity of the nuclear soluble, nuclear chromatin-bound, and cytosolic fractions, respectively. Both maspin^WT^ and maspin ^D346E^ were present in the cytosolic compartment. However, a higher level of maspin^D346E^, as compared to maspin^WT^, was detected in the nuclear soluble fraction. Moreover, both nuclear maspin^WT^ and nuclear maspin^D346E^ were detected in the chromatin-bound fractions.

DU145 cells infected with adenovirus expressing either maspin^WT^ or maspin^D346E^ were fixed and subjected to maspin immunofluorescence staining (green) and confocal microscope imaging. **Figure 2A** shows that maspin^WT^ was predominantly localized in the cytoplasm. In contrast, maspin^D346E^ was completely within the nuclear envelope as judged by the staining of lamin B (red). To confirm that the nuclear localization of maspin^D346E^ was not cell line specific, lung cancer cell line H1299, which expresses no detectable level of maspin (**Figure 2B**), was infected by adenovirus. Similarly to the results with DU145 cells, maspin^WT^ was localized in the cytoplasm, whereas maspin^D346E^ was predominantly localized in the nuclei of H1299 cells (**Figure 2C**).

To ascertain the differential subcellular distributions of maspin^D346E^ and maspin^WT^, H1299 cells expressing recombinant maspin^WT^ or maspin^D346E^ were fractionated. As shown in **Figure 2D**, western blotting of HDAC1, histone 3, and Hsp90 plus GAPDH, demonstrated the purity of the nuclear soluble, nuclear chromatin-bound, and cytosolic fractions, respectively. Both maspin^WT^ and maspin ^D346E^ were present in the cytosolic compartment. However, a higher level of maspin^D346E^, as compared to maspin^WT^, was detected in the nuclear soluble fraction. Moreover, both nuclear maspin^WT^ and nuclear maspin^D346E^ were detected in the chromatin-bound fractions.

To quantify maspin protein in each subcellular compartment, it is important to account for maspin in all possible molecular contexts, which may not be recapitulated by the western blot of the monomeric maspin. For this reason, fractionated cellular proteins from infected H1299 cells were subjected to IP with maspin antibody and MS. Spectral counting showed no maspin peptides in the IP control with pre-immune IgG. Maspin^WT^ was only found in the cytosolic compartment with identification of 4 unique peptides and 5 total spectra. In parallel, maspin ^D346E^ was found both in the nuclear compartment with the identification of 11 unique peptides and 15 total spectra, and in the cytosolic fraction with the identification of 6 unique peptides and 7 total spectra. Sequence coverage of maspin is shown in **Figure S1** (The differential subcellular localizations of maspin^WT^ and maspin^D346E^ were statistically significant (p<0.01, Fisher Exact Test). Considering that the maspin-containing multi-protein complex might be dissociated if the stringency of the cell fraction was high, an independent cell fractionation was performed at lower dissociation stringency. MS/MS detected 12 unique and 648 total histone spectra counts in the nuclear fraction; and 6 unique and 68 total histone spectra counts in the cytosolic fraction (**Table S1**), demonstrating the effective separation of nuclear and cytosolic fractions by this method. Consistently, maspin^WT^ was found exclusively in the cytosolic fraction whereas maspin^D346E^ was identified in both the cytosol (90% of spectra) and in the nucleus (10% of spectra). We speculate that the less stringent nuclear extraction may have preserved the tighter protein-protein interaction and reduced the efficiency of the IP of free maspin. Taken together, these data confirmed further enrichment of maspin^D346E^ in the nucleus, as compared to the predominant cytosolic presence of maspin^WT^.

### Dominant Nuclear Localization of Maspin^D346E^ in Cells that Naturally Distribute Maspin to Cytoplasm and Nucleus

**Figure 3:**
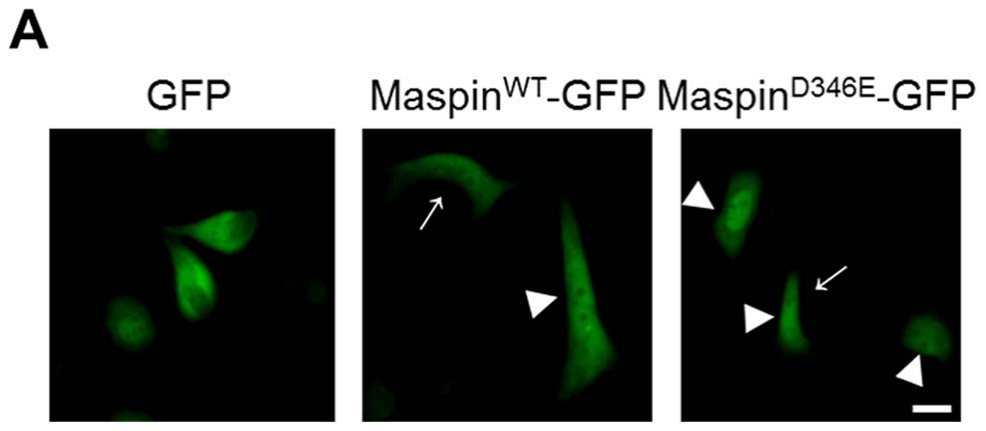
Dominant nuclear localization of maspin^D346E^ in cells that naturally distribute maspin to cytoplasm and nucleus. (**A**) Immunofluorescent imaging of live PC3 cells 48 hrs after transient transfection with pEGFP N1-maspin^WT^ and pEGFP N1-maspin^D346E^ → indicates cytosolic maspin whereas ▸ indicates nuclear maspin. pEGFP was used as a control. Scale bar = 20 µm.

It is noted that when expressed in low grade adenocarcinoma and cancer cell lines, maspin is always distributed into the cytoplasm and the nucleus. To test whether the subcellular distribution of maspin^D346E^ is distinct as compared to maspin^WT^ in a cancer cell line that naturally expresses and distributes maspin, we utilized the prostate cancer cell line PC3 [7]. In an effort to differentiate between endogenous and recombinant maspin in PC3 cells, PC3 cells were transiently transfected to express maspin^WT^-GFP or maspin^D346E^-GFP. The GFP alone was used as a negative control. **Figure 3A** shows fluorescence imaging of live PC3 cells 48 hrs after the transfection. While the GFP alone exhibited a mixed cytosolic plus nuclear distribution as expected based on the published records [36]–[38], the maspin^WT^-GFP was equally distributed to cytoplasm and nucleus of each maspin-expressing cell, reminiscent of the natural distribution of endogenous maspin in PC3 cells. In contrast, maspin^D346E^ was particularly enriched in the nucleus (75% of the cells). These data suggest that D^346^ may act as a dominant *cis* element for maspin nuclear localization.

### Nuclear Localization of Maspin^WT^ but not Maspin^D346E^ is Inhibited by Nuclear Export Inhibitor Leptomycin B (LMB)

**Figure 4:**
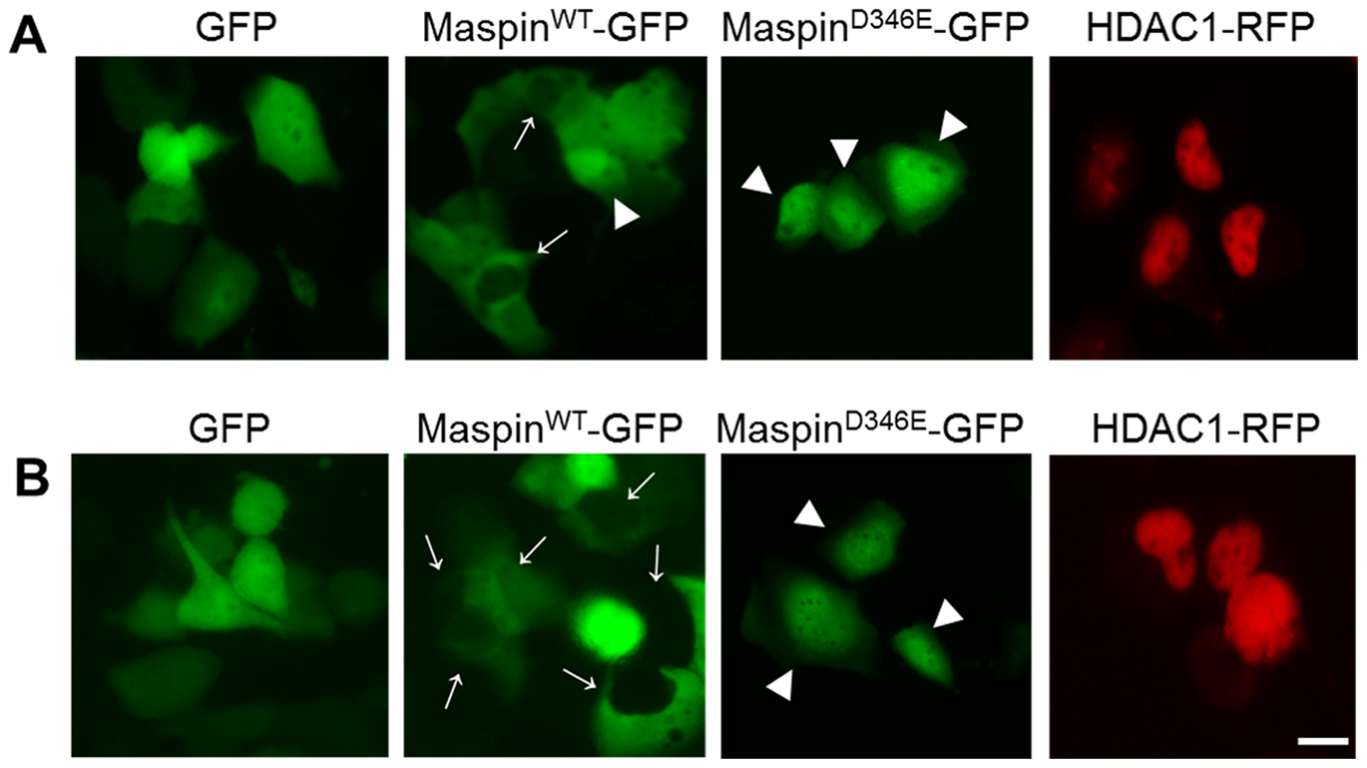
Nuclear localization of maspin^WT^ but not maspin^D346E^ is inhibited by nuclear export inhibitor Leptomycin B (LMB). (**A**) Immunofluorescence imaging (x40) of live H1299 cells 40 hrs after transient transfection with pEGFP N1-maspin^WT^ and pEGFP N1-maspin^D346E^. → indicates cytosolic maspin whereas ▸ indicates nuclear maspin. pEGFP N1 and was used as a control. (**B**) Immunofluorescence imaging of live H1299 cells 44 hrs after transient transfection with pEGFP N1-maspinWT and pEGFP N1-maspin^D346E^ in the presence of LMB (2.5 ng/mL, 4 hrs). → indicates cytosolic maspin whereas ▸ indicates nuclear maspin. Expression of GFP alone or HDAC1-RFP was used as controls for (**A**) and (**B**). Scale bar = 20 µm.

To date, there are no known inhibitors of nuclear import. To examine whether the increased accumulation of nuclear maspin^D346E^ was a result of the deficiency or absence of its nuclear export, transiently transfected H1299 cells expressing either maspin^WT^-GFP or maspin^D346E^-GFP were treated with LMB, a potent inhibitor of CRM1 (chromosomal region maintenance/exportin 1), a protein required for nuclear export of proteins containing NES [39]–[41]. Under LMB treatment, molecules that depend on CRM1 export from the nucleus, such as HDAC1, are expected to be accumulated in the nuclei [42]. The essence of using live imaging was based on the need to capture LMB-induced temporal sequence of maspin trafficking. As shown in **Figure 4**, to our surprise, LMB treatment of transiently transfected H1299 cells led to significant reduction of nuclear maspin^WT^-GFP, and had little effect on the nuclear localization of maspin^D346E^-GFP. It is likely that maspin trafficking was not directly controlled at the step of nuclear export. Instead, our data suggest that the nuclear retention of maspin^WT^, but not maspin ^D346E^, may be competitively inhibited by a yet-to-be-identified factor (Factor X) that is exported by a CRM1-dependent mechanism and therefore sensitive to LMB treatment. An LMB-led nuclear accumulation of Factor X may consequently displace nuclear maspin. Collectively, this data demonstrates the importance of a single amino acid D346 in controlling the molecular context and nuclear retention of maspin.

### Maspin Subcellular Localization does not Involve Endoplasmic Reticulum (ER) Retention

**Figure 5:**
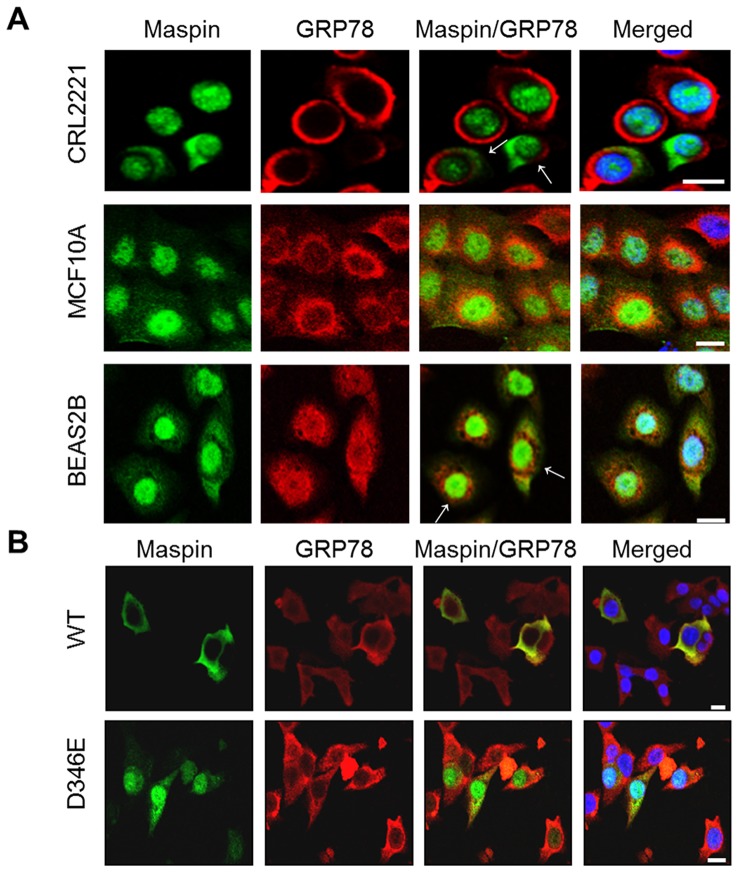
Maspin subcellular localization does not involve ER retention. (**A**) Confocal immunofluorescence imaging of endogenous maspin (green) and GRP78 (red) in CRL2221, MCF10A and BEAS2B cells. Nuclei were counterstained with DAPI. (**B**) Confocal immunofluorescence imaging of recombinant maspin (green) and endogenous GRP78 (red) in DU145 cells. Nuclei were counterstained with DAPI. Scale bars = 20 µm.

Upon careful study of maspin protein sequence, we have recognized that maspin D^346^ is within a unique intramolecular KDEL motif (K^345^–L^348^), which, if located at the very C-terminal of a protein, is also known as an endoplasmic reticulum (ER) retrieval signal [43], [44]. To test the possibility that the intramolecular KDEL motif may still play a role in maspin trafficking, we profiled maspin subcellular localization by immunofluorescence staining in three normal immortalized cell lines from prostate (CRL2221), breast (MCF10A) and lung (BEAS 2B). As shown in **Figure 5A**, the presence of predominantly nuclear localization of maspin (green), with some cytosolic presence, was shown in early passage CRL2221 and MCF10A cells and it was confirmed in BEAS2B cells [19]. It is worth noting that maspin subcellular distribution between the nucleus and cytoplasm became more heterogeneous as the cell culture passage number increased (data not shown). This observation was consistent with an earlier reports that maspin produced by MCF10A cells was both nucleo-cytoplasmic [45] and secreted [46] where the amount of secreted maspin depended on cell passage. Regardless, the cytosolic maspin expressed by these three cell lines did not co-localize with glucose regulated protein 78 (GRP78, red), an ER marker. Interestingly, ER function, as well as the regulation of ER-resident proteins, has been shown to be differentially regulated in tumor progression [47]–[50]. To test whether tumor cells that distribute recombinant maspin^WT^ and maspin^D346E^ with distinct subcellular patterns (as compared to normal cells) were affected by ER function, prostate tumor cells DU145 infected by adenovirus encoding these two proteins, respectively, were analyzed by immunofluorescence staining in parallel (**Figure 5B**). Not surprisingly, it was shown that the GRP 78 (red) immunofluorescence staining in DU145 cells was dispersed throughout the cytoplasm. Maspin^WT^ was perinuclear and cytosolic whereas maspin^D346E^ was predominantly nuclear. While GRP78 seemed to be an effector of ER function, it is unlikely that maspin trafficking is regulated directly by an ER-dependent mechanism.

### Increased Nuclear Localization of Maspin Correlates with Increased HDAC1 Interaction

**Figure 6:**
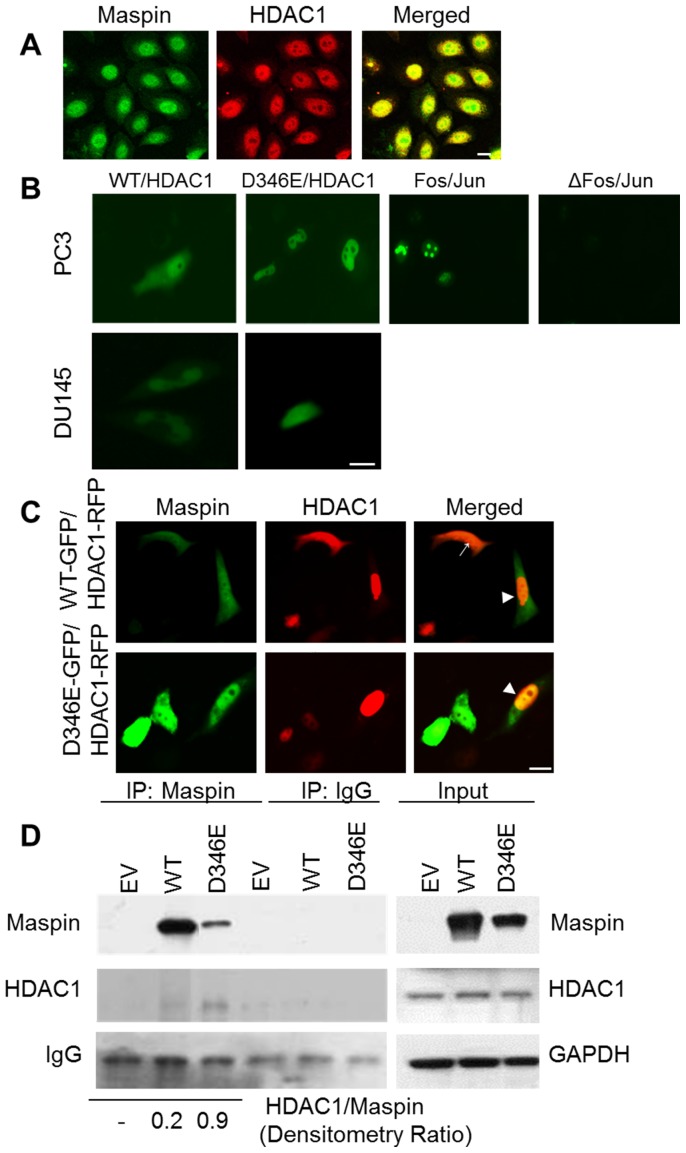
Increased nuclear localization of maspin^D346E^ correlates with increased HDAC1 interaction. (**A**) Confocal imaging of endogenous maspin (green) and HDAC1 (red) immunofluorescence staining in MCF10A cells. Scale bar = 20 µm (**B**). Bimolecular fluorescence complementation (BIFC) of live PC3 and DU145 cells transiently transected with pBiFC maspin^WT^ YC or pBiFC maspin^D346E^ YC in combination with pBiFC HDAC1 YN. The BiFC of bJunYN and BiFC bFosYC and BiFC of bJunYN and BiFC bFosYC Δ179–193 were used as positive and negative controls, respectfully, in PC3 cells. Scale bar = 10 µm (**C**) Immunofluorescence imaging (x40) of live PC3 cells after co-transfection with either pEGFP N1-maspin^WT^ or pEGFP N1 maspin^D346E^ in combination with pcDNA3.1 HDAC1-RFP. → indicates cytosolic maspin/HDAC1 interaction and ▸ indicates nuclear maspin/HDAC1 interaction. Scale bar = 20 µm. (**D**) Western blot of recombinant maspin and HDAC1 after immunoprecipitation (IP) with maspin antibody in DU145 cells. The mouse IgG was used as a negative control. Total levels of maspin, HDAC1, and loading control GAPDH are shown in the input panel. Numbers below represent normalized HDAC1/maspin ratio.

HDAC1 is predominantly a nuclear protein under normal physiological conditions. However, it was noted that under pathological conditions it could be translocated to the cytoplasm [42]. To date, maspin remains the only polypeptide inhibitor of HDAC1 identified. It is important to examine whether the differential trafficking of maspin is, at least in part, accompanied by a consistent trafficking pattern of HDAC1. As shown in **Figure 6A**, in normal immortalized epithelial cells MCF10A, maspin was primarily co-localized with HDAC1 in the nucleus, suggesting the association of maspin with HDAC1.

To test whether HDAC1 and maspin directly interact with each other, we utilized the bimolecular fluorescence complementation (BiFC) [33], [51], a method that detects the signal of protein-protein interaction only when the two proteins make physical contact. We expressed maspin-YC fusion protein and HDAC1-YN fusion protein simultaneously in PC3 and DU145 cells by double transient transfections. As shown in **Figure 6B**, both maspin^WT^ and maspin^D346E^ directly interacted with HDAC1 as evident by green fluorescence. Interestingly, while the interaction of maspin^WT^ and HDAC1 occurred in the nucleus and the cytoplasm, a robust interaction of maspin^D346E^ with HDAC1 occurred almost exclusively in the nucleus. The interaction of Fos/Jun and ΔFos/Jun in the nucleus was used as positive and negative controls, respectfully, for the BiFC constructs. In parallel, we also utilized the live fluorescence imaging of GFP-tagged maspin and RFP tagged HDAC1 co-expressed in PC3 cells. As shown in **Figure 6C**, co-localization of maspin^WT^-GFP and HDAC1-RFP was detected in both, the nucleus and the cytoplasm, in accordance with natural expression of maspin in PC3 cells. In contrast, increased nuclear localization of HDAC1-RFP correlated with increased nuclear interaction of maspin^D346E^-GFP and HDAC1-RFP. Consistent results were obtained from IP-MS proteomic analysis. As shown in **Figure 6D**, not only that maspin^D346E^ was able to interact with endogenous HDAC1 but more HDAC1 co-precipitated with maspin^D346E^ relative to the maspin^WT^ (HDAC1 to maspin densitometry ratio 0.2 *vs.* 0.9 respectively). These data suggest that maspin^D346E^ may have greater affinity towards HDAC1 as compared to maspin^WT^.

### Maspin Nuclear Localization Correlates with Increased Histone Acetylation and Release of HDAC-repressed Gene Expression

**Figure 7:**
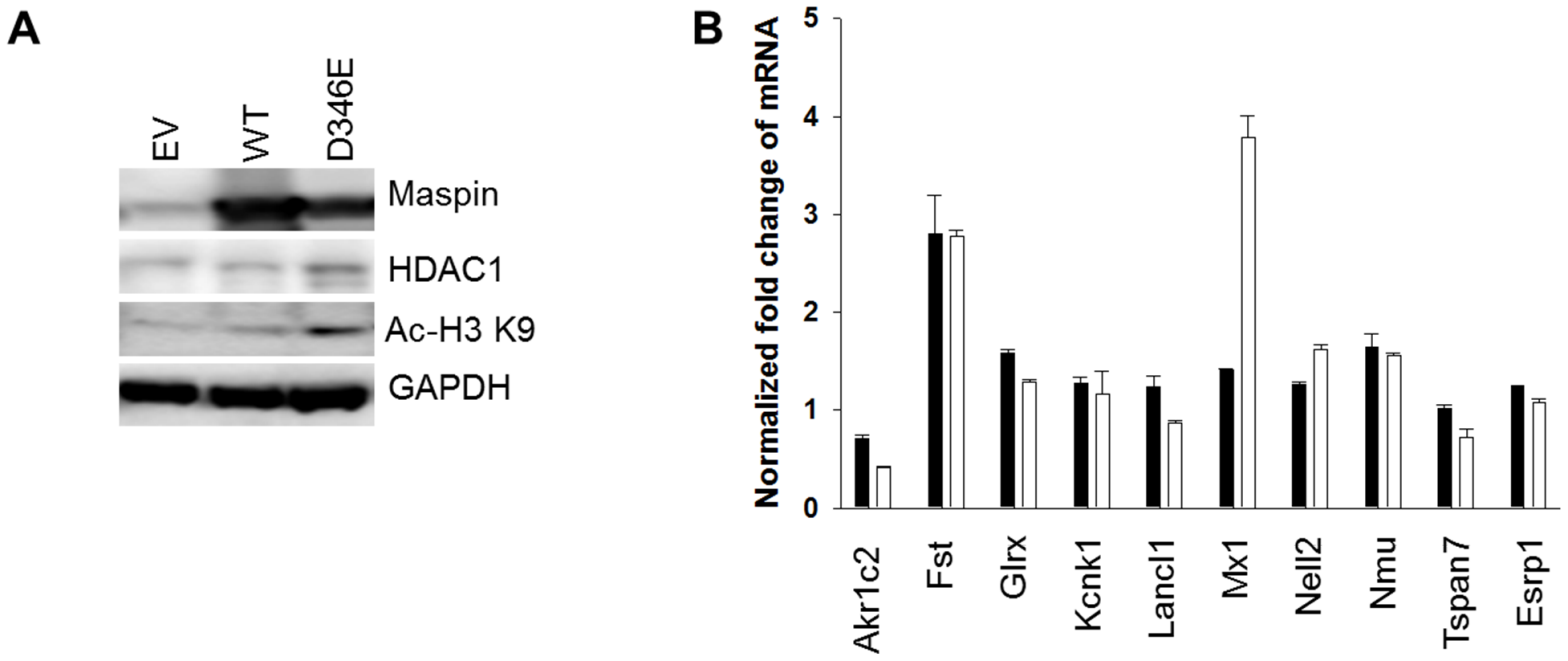
Maspin nuclear localization correlates with increased histone acetylation and release of HDAC-repressed gene expression. (**A**) Western blot of recombinant maspin, HDAC1, and HDAC1 target protein (acetylated Histone 3 at Lysine 9 (H3 Acetyl K^9^)) in DU145 cells. GAPDH was used as a loading control. (**B**) Q-RT-PCR of HDAC1 targeted genes differentially regulated by maspin. The threshold cycle (ct) numbers obtained from qRT-PCR were normalized by the internal GAPDH controls and presented as the fold change. Maspin^WT^: black bar; maspin^D346E^: white bar.

To date, maspin remains the only known endogenous polypeptide inhibitor of HDAC1. If maspin^D346E^ had a similar (or higher) affinity to HDAC1 as maspin^WT^, increased nuclear abundance of maspin^D346E^ may lead to increased level of histone acetylation and up-regulation of HDAC1 target gene expression. Indeed, as compared to maspin^WT^, maspin^D346E^ expression, *via* adenoviral infection, in DU145 cells led to a greater level of histone 3 (H3)-Lysine 9 (K^9^) acetylation (**Figure 7A**). To determine whether the correlation of maspin^D346E^ with increased H3- K^9^ acetylation was consequential in the up-regulation of HDAC1 target genes, the expression level of 10 HDAC1 target genes known to be up-regulated in the presence of maspin^WT^ was assessed by q-RT-PCR [12]. As shown in **Figure 7B**, 2 out of 10 HDAC1 target genes tested (Interferon-induced GTP-binding protein Mx1 and protein kinase C-binding protein NELL2) showed further increase in their mRNA expression in the presence of maspin^D346E^ (not statistically significant). Taken together, these data suggest, that maspin^D346E^ may function as a stronger HDAC1 inhibitor, with increased affinity for HDAC1 and increased nuclear compartmentalization.

## Discussion

In this study, using the mutagenesis approach we identified amino acid residue D^346^ as a unique intrinsic element of maspin that can be manipulated to redirect maspin from the cytoplasm to the nucleus, accompanied with increased inhibitory effect of maspin on HDAC1 in tumor cells. This conclusion is substantiated by our evidence derived with multiple cancer cell lines from prostate and lung, with and without natural maspin expression (DU145, PC3 and H1299), and with multiple experimental approaches to re-express intact or fusion maspin proteins (adenoviral infections, transient transfections and BiFC).

This study extends from our earlier evidence that maspin acts as an endogenous HDAC1 inhibitor. While the shift in the subcellular localization of maspin from the nucleus to the cytoplasm during cancer progression may be an early marker for tumor progression, HDAC1 is also shown to be differentially regulated during tumor progression [52]. Under pathological conditions, such as cancer, the overall expression level of HDAC1 is increased. Furthermore, HDAC1 presence and activity are noted in the cytoplasm contributing to the activity and function of certain tumor promoting molecules such as HSP90 [53]. It remains unclear whether the translocation of maspin from the nucleus to the cytoplasm in tumor progression is an adaptation in response to the adverse HDAC1 activity in the cytoplasm. Nonetheless, the ultimate loss of maspin expression in high grade carcinoma will contribute to yet an additional increase of HDAC1 activity, on top of the increased HDAC1 expression, thus promoting tumor progression.

Previously, we demonstrated that either purified or endogenously expressed maspin is bound to and inhibits HDAC1 [7]. Data from the current study confirmed the nuclear interaction of endogenous maspin and endogenous HDAC1 in normal epithelial cells. Considering the multi-component nature and the complex dynamics of the HDAC1 complex [54], the possibility that maspin may actually directly bind to another component in the same HDAC1 complex cannot be unambiguously excluded. Nonetheless, evidence presented in the current study is consistent with the structural consideration [55]–[57] that maspin may indeed directly interact with HDAC1. Further in line with this evidence, functional consequence of maspin/HDAC1 interaction and inhibition has been demonstrated previously by our lab, suggesting that through HDAC1 inhibition, maspin controls a small set of genes involved in epithelial differentiation [12]. Subsequently Lee *et al*. demonstrated that maspin inhibition of HDAC1 increased acetylation of HDAC1 target protein Ku70 resulting in an increase in apoptosis [58]. In addition to directly blocking the HDAC1-mediated gene repression and deacetylation, maspin may also play a role in dis-coupling the steps of histone modification and DNA modification, and preventing pathological DNA silencing. Our earlier study showed the effect of maspin in reversing pathological DNA methylation-silencing of glutathione S-transferase pi (GSTp), a tumor suppressor implicated in prostate cancer [59]. As compared to pharmacological HDAC inhibitors, the inhibition of HDAC1 by maspin may be more specific, as well as more coordinated in concert with other mechanisms involved in epithelial differentiation. Indeed, while maspin up-regulated HDAC1 target genes, it also led to decreased expression of a specific cluster of genes closely associated with TGFβ signaling [12], which plays a key role in epithelial cell dedifferentiation.

**Figure 8:**
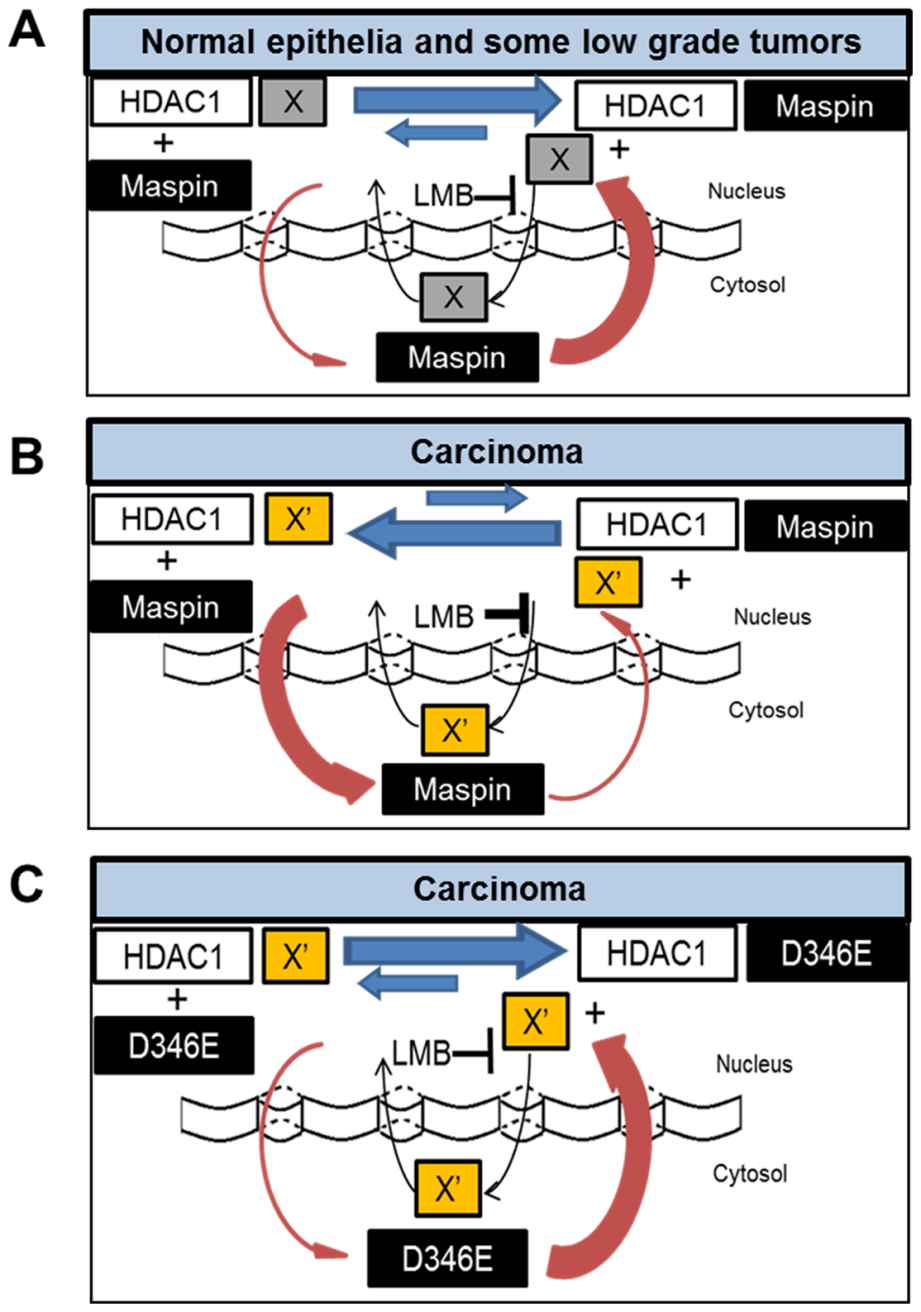
A hypothetical model explaining disregulation of maspin in tumor progression. (**A**) Benign tumor or better differentiated cancer with better prognosis is associated with nuclear maspin. Nuclear maspin has a stronger affinity towards HDAC1 as compared to hypothetical HDAC1-associated nuclear factor X. (**B**) High grade carcinoma exhibiting a less differentiated phenotype and worse prognosis is associated with cytosolic maspin or loss of maspin. In cancer, hypothetical HDAC1- associated nuclear factor X is X′ and it has a stronger affinity towards HDAC1 as compared to maspin. (**C**) Therapeutic potential of bioengineered maspin mutant or its derivative in treating advanced disease. Maspin mutant restores maspin nuclear localization and HDAC1 inhibition in advanced disease.

The subcellular localization of maspin does not appear to be regulated by the classical nuclear exclusion pathway or as a direct consequence of HDAC1 subcellular distribution. Instead, data from the current study support a novel hypothetical model as illustrated in **Figure 8**. According to this model, maspin may co-exist with another HDAC1-binding factor (X) that is yet to be identified, for HDAC1. Normal epithelial tissue, benign tumor or better differentiated carcinoma with better prognosis and better overall patient survival are associated with nuclear maspin that has a stronger affinity towards HDAC1 than factor X (**Figure 8A**). This stronger affinity of nuclear maspin towards HDAC1 translates into maspin-specific HDAC1 inhibition and upregulation of a small subset of HDAC1 target genes involved in the regulation of epithelial differentiation [12]. Additionally, since the majority of maspin may be HDAC1-associated, the net efflux of maspin favors nuclear localization. **Figure 8B** may depict the scenario of high grade carcinoma which is characterized by less differentiated phenotype, worse patient prognosis, cytosolic maspin or, in certain cases, the complete loss of maspin. Under these circumstances, the hypothetical HDAC1- associated nuclear factor X may be replaced by X′, which displays stronger affinity towards HDAC1 and competes against maspin for nuclear retention [14].

Our experimental evidence suggests that where maspin^WT^ fails to compete against X′ for HDAC1 in the nucleus of poorly differentiated tumor cells, subtle differences in maspin RCL and its neighboring regions, such as maspin^D346E^, may restore the nuclear presence (**Figure 8C**) as well as the nuclear activity of maspin, and ultimately restore epithelial differentiation [12]. This model helps explain why, as compared to maspin^WT^ that was almost completely excluded from the nucleus upon LMB treatment, the nuclear localization of maspin^D346E^ was not affected by LMB treatment. Further, based on our data and hypothetical model, the nuclear presence of maspin^D346E^ may be dominant even in tumor cells that have already acquired the disregulated subcellular distribution of endogenous maspin. As compared to many other tumor suppressor genes such as p53 that are frequently mutated with numerous hotspots, naturally occurring maspin, with the exception of a couple of popular polymorphisms, is rarely mutated in tumor progression [60]. Thus, a maspin-mimetic drug, as exemplified by maspin^D346E^, may not suffer from significant interference by the background level of endogenous maspin.

It is well established that tumor cells may be sensitive to the toxicity of HDAC-targeted therapies [61]. While broad-spectrum HDAC inhibitors that target all HDACs in all subcellular compartments have a number of adverse side effects, it is intriguing to postulate that isoform-specific HDAC inhibitors that can be delivered to the correct subcellular compartment may improve the efficacy of the drugs. Lesson learned from maspin revealed the complexity and versatility of endogenous HDAC inhibitor that are not fully recapitulated by synthetic pharmacological HDAC inhibitors in clinical studies [62], [63]. Our experimental evidence with maspin^D346E^ raised the importance of maspin molecular partnership that can be dictated by maspin sequence and may be used as the basis for better design of maspin-mimetic HDAC1-targeted anti-cancer drugs.

In summary, our findings and hypothetical model provide novel insights regarding the regulation and functional attributes of maspin translocation from the nucleus to the cytoplasm in tumor progression. The identification of a critical amino acid residue in the maspin RCL is likely to open a new window of opportunity for the development of maspin-based biologically competent HDAC inhibitor for cancer treatment.

## Supporting Information

Figure S1 **Sequence coverage of maspin in gel slices from samples isolated from nuclei.** Highlighted yellow indicates a peptide containing identified amino acids. Highlighted in green indicates posttranslational modified amino acids. The modification of methionine was oxidation, +16 daltons. Five maspin spectra and corresponding peptide sequence is shown.
(TIF)

Table S1 **Quantification of histones in the nuclear and cytosolic preparations based on total spectral counts.**
(PPTX)
