# Supplementary figures and images for: Identification of an Intrinsic Determinant Critical for Maspin Subcellular Localization and Function
Source: PLoS One. 2013 Nov 21;8(11):e74502. doi: 10.1371/journal.pone.0074502 (PMC3837015; doi:10.1371/journal.pone.0074502)

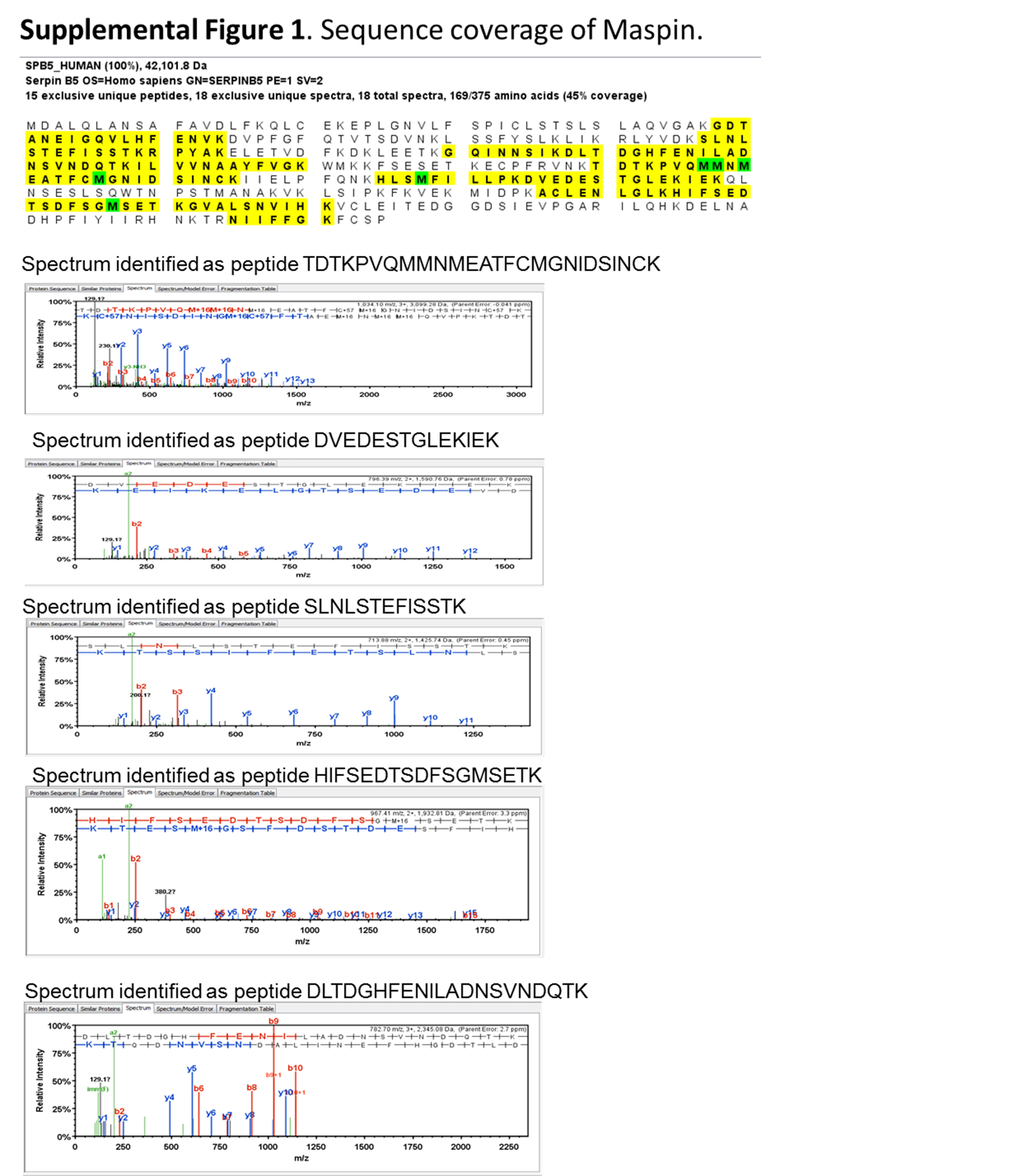

Supplement: Figure S1 — Sequence coverage of maspin in gel slices from samples isolated from nuclei. Highlighted yellow indicates a peptide containing identified amino acids. Highlighted in green indicates posttranslational modified amino acids. The modification of methionine was oxidation, +16 daltons. Five maspin spectra and corresponding peptide sequence is shown. (TIF) [file pone.0074502.s001.tif]
