# Supplementary material for: Identification of an Intrinsic Determinant Critical for Maspin Subcellular Localization and Function
Source: PLoS One. 2013 Nov 21;8(11):e74502. doi: 10.1371/journal.pone.0074502 (PMC3837015; doi:10.1371/journal.pone.0074502)
Supplement: Table S1 — Quantification of histones in the nuclear and cytosolic preparations based on total spectral counts. (PPTX) [file pone.0074502.s002.pptx]

## Slide 1
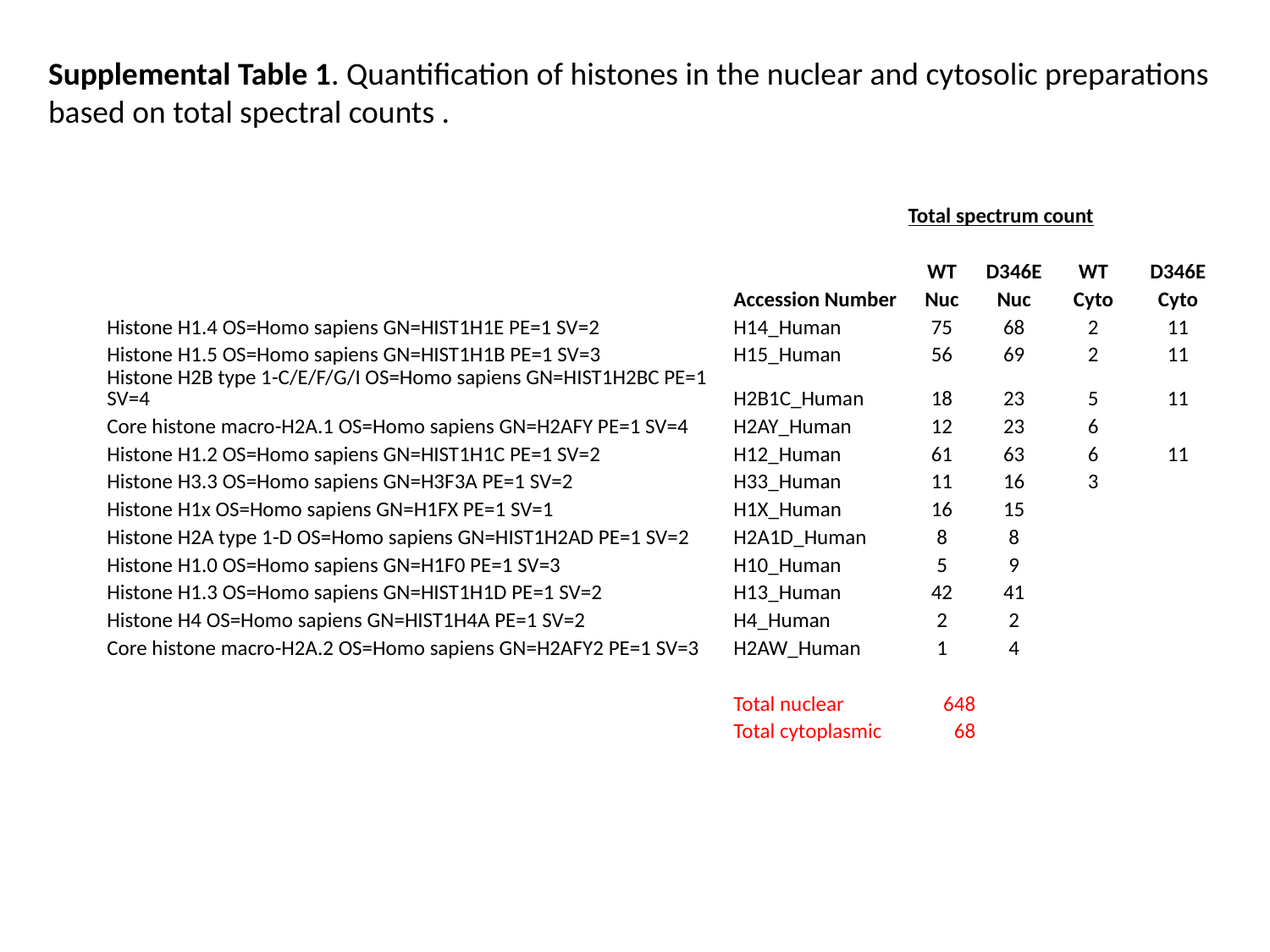

Supplemental Table 1. Quantification of histones in the nuclear and cytosolic preparations
based on total spectral counts .
| | | | | | | | | | Total spectrum count | | | |
| --- | --- | --- | --- | --- | --- | --- | --- | --- | --- | --- | --- | --- |
| | | | | | | | | | | | | |
| | | | | | | | | | WT | D346E | WT | D346E |
| | | | | | | | | Accession Number | Nuc | Nuc | Cyto | Cyto |
| Histone H1.4 OS=Homo sapiens GN=HIST1H1E PE=1 SV=2 | | | | | | | | H14\_Human | 75 | 68 | 2 | 11 |
| Histone H1.5 OS=Homo sapiens GN=HIST1H1B PE=1 SV=3 | | | | | | | | H15\_Human | 56 | 69 | 2 | 11 |
| Histone H2B type 1-C/E/F/G/I OS=Homo sapiens GN=HIST1H2BC PE=1 SV=4 | | | | | | | | H2B1C\_Human | 18 | 23 | 5 | 11 |
| Core histone macro-H2A.1 OS=Homo sapiens GN=H2AFY PE=1 SV=4 | | | | | | | | H2AY\_Human | 12 | 23 | 6 | |
| Histone H1.2 OS=Homo sapiens GN=HIST1H1C PE=1 SV=2 | | | | | | | | H12\_Human | 61 | 63 | 6 | 11 |
| Histone H3.3 OS=Homo sapiens GN=H3F3A PE=1 SV=2 | | | | | | | | H33\_Human | 11 | 16 | 3 | |
| Histone H1x OS=Homo sapiens GN=H1FX PE=1 SV=1 | | | | | | | | H1X\_Human | 16 | 15 | | |
| Histone H2A type 1-D OS=Homo sapiens GN=HIST1H2AD PE=1 SV=2 | | | | | | | | H2A1D\_Human | 8 | 8 | | |
| Histone H1.0 OS=Homo sapiens GN=H1F0 PE=1 SV=3 | | | | | | | | H10\_Human | 5 | 9 | | |
| Histone H1.3 OS=Homo sapiens GN=HIST1H1D PE=1 SV=2 | | | | | | | | H13\_Human | 42 | 41 | | |
| Histone H4 OS=Homo sapiens GN=HIST1H4A PE=1 SV=2 | | | | | | | | H4\_Human | 2 | 2 | | |
| Core histone macro-H2A.2 OS=Homo sapiens GN=H2AFY2 PE=1 SV=3 | | | | | | | | H2AW\_Human | 1 | 4 | | |
| | | | | | | | | | | | | |
| | | | | | | | | Total nuclear | 648 | | | |
| | | | | | | | | Total cytoplasmic | 68 | | | |
